# Ceramide Induces Human Hepcidin Gene Transcription through JAK/STAT3 Pathway

**DOI:** 10.1371/journal.pone.0147474

**Published:** 2016-01-25

**Authors:** Sizhao Lu, Sathish Kumar Natarajan, Justin L. Mott, Kusum K. Kharbanda, Duygu Dee Harrison-Findik

**Affiliations:** 1 Department of Biochemistry and Molecular Biology, University of Nebraska Medical Center, 985870 Nebraska Medical Center, Omaha, NE, 68198–5870, United States of America; 2 Division of Gastroenterology and Hepatology, Department of Internal Medicine, University of Nebraska Medical Center, 982000 Nebraska Medical Center, Omaha, NE, 68198–2000, United States of America; INRA, FRANCE

## Abstract

Changes in lipid metabolism and iron content are observed in the livers of patients with fatty liver disease. The expression of hepcidin, an iron-regulatory and acute phase protein synthesized by the liver, is also modulated. The potential interaction of lipid and iron metabolism is largely unknown. We investigated the role of lipid intermediate, ceramide in the regulation of human hepcidin gene, *HAMP*. Human hepatoma HepG2 cells were treated with cell-permeable ceramide analogs. Ceramide induced significant up-regulation of *HAMP* mRNA expression in HepG2 cells. The effect of ceramide on *HAMP* expression was mediated through transcriptional mechanisms because it was completely blocked with actinomycin D treatment. Reporter assays also confirmed the activation of 0.6 kb *HAMP* promoter by ceramide. HepG2 cells treated with ceramide displayed increased phosphorylation of STAT3, JNK, and NF-κB proteins. However, ceramide induced the binding of STAT3, but not NF-κB or c-Jun, to *HAMP* promoter, as shown by the chromatin immunoprecipitation assays. The mutation of STAT3 response element within 0.6 kb *HAMP* promoter region significantly inhibited the stimulatory effect of ceramide on *HAMP* promoter activity. Similarly, the inhibition of STAT3 with a pan-JAK kinase inhibitor and STAT3 siRNA pool also diminished the induction of both *HAMP* promoter activity and mRNA expression by ceramide. In conclusion, we have shown a direct role for ceramide in the activation of hepatic *HAMP* transcription via STAT3. Our findings suggest a crosstalk between lipid and iron metabolism in the liver, which may contribute to the pathogenesis of obesity-related fatty liver disease.

## Introduction

More than one third of the US adult population is estimated to have non-alcoholic fatty liver disease (NAFLD) [1] and its prevalence is further expanding to both developing countries and children [2]. NAFLD, by definition, is the accumulation of fat (steatosis) in the livers of patients with no or little alcohol consumption [3]. Inflammation (steatohepatitis) and iron are important secondary risk factors for the progression of NAFLD to non-alcoholic steatohepatitis (NASH), which can eventually lead to cirrhosis and hepatocellular carcinoma [4–7]. NAFLD/NASH patients frequently display elevated levels of serum iron indices and hepatic iron content [8,9]. The deposition of iron in the liver correlates with disease severity and the development of fibrosis [10–12].

The discovery of hepcidin was instrumental in understanding the connection between inflammation and iron homeostasis [13–16]. Hepcidin, mainly synthesized in the hepatocytes of the liver, is both an acute phase protein and a pivotal regulator of iron metabolism [13–16]. Hepcidin controls systemic iron homeostasis by inhibiting the function of iron exporter ferroportin [17]. As an acute phase protein, hepcidin responds to inflammation. Hepcidin expression is hence regulated by the inflammatory cytokines, IL-1 and IL-6. The transcription of hepcidin gene, *HAMP* has been shown to be activated by the binding of the transcription factor, STAT3 to *HAMP* promoter [14,15,18]. STAT3 is regulated by Janus kinases (JAK) via phosphorylation of a conserved tyrosine residue near the C-terminus [19].

Several factors including increased gut permeability, adipose tissue-derived cytokines and adipokines, Kupffer cell activation, and lipid accumulation induce inflammation in the livers of NAFLD patients [7,20]. Hepcidin expression is modulated in NAFLD patients [12,21,22] but the underlying mechanisms are unclear. In overly obese patients undergoing bariatric surgery, a relationship between adipose tissue-derived IL-6, and elevated hepcidin expression in adipose tissue has been shown [22].

Fat accumulation and inflammation in the livers of NAFLD patients stimulate the synthesis of the sphingolipid, ceramide through de novo and sphingomyelinase pathways [23,24]. Accordingly, elevated hepatic fat content is associated with increased ceramide levels in obese individuals [25] while weight loss is correlated with reduced pro-ceramide gene expression, decreased serum ceramide levels, and the reversal of NASH pathogenesis in the liver [26]. Animal studies have also demonstrated that high fat intake increases ceramide production in the liver [27] and the inhibition of ceramide synthesis reduces hepatic steatosis and improves insulin signaling [28,29]. The mechanisms by which ceramide participates in the pathogenesis of NAFLD and the regulation of *HAMP* expression is unknown.

Although initially regarded simply as a structural component of biomembranes, ceramide has recently been recognized as a signaling molecule [24]. Besides its negative effect on insulin signaling [30], ceramide also activates inflammatory signaling pathways. Both the tyrosine phosphorylation and DNA-binding activity of the transcription factor, STAT3 has been shown to be stimulated by ceramide in a JAK-dependent manner in cultured fibroblast cells [31]. Ceramide has also been reported to activate the transcription factor, NF-κB and its down-steam targets in HepG2 and other cells [32–34]. A role for c-Jun N-terminal kinase (JNK) has been shown both in inflammation and steatosis in NAFLD [35]. The absence of JNK1 expression in a knockout mouse model has been shown to inhibit steatohepatitis induced by methionine-choline deficient diet intake [36]. Upon phosphorylation and activation, JNK in turn phosphorylates serine residues in the N-terminal of c-Jun and thereby up-regulates its capability to trans-activate genes harboring AP-1 enhancer sequences in the promoter region [37]. Ceramide has also been reported to stimulate JNK activation [38–40]. Despite being regulated by metabolic changes and inflammation, and its direct participation in inflammatory signaling, the effect of ceramide on hepatic *HAMP* expression is unknown. In the current study, we investigated the mechanisms underlying *HAMP* regulation by ceramide in human hepatoma cells.

## Materials and Methods

### Reagents

Synthetic cell-permeable ceramide analogs, C2 (#62525), and C6 (#62510) ceramide, and pan-JAK kinase inhibitor, JAK inhibitor I (#15146) were purchased from Cayman Chemical. JNK inhibitor, SP600125 was purchased from Enzo Life Sciences. siGENOME Human STAT3 siRNA SMART pool and control siRNA were obtained from Dharmacon.

### Cell culture

HepG2 human hepatoma cells, obtained from the American Type Culture Collection (ATCC), were maintained in high glucose Dulbecco’s modified Eagle’s medium (DMEM) supplemented with glutamine and 10% fetal calf serum (Atlantic Biologicals). 1.3×10^6^ or 3.9 ×10^6^ cells were seeded in 25 cm^2^ or 75 cm^2^ flasks, respectively overnight prior to experiments. Cells were treated with ceramide analogs or solvent (0.1% DMSO), as control, for 8 hours. All inhibitors were added 1 hour prior to treatments with ceramide or DMSO.

### Transfections

Lipofectamine 3000 (Life Technologies) was employed for the transfection of plasmids for dual luciferase assays. RNAiMax (Life Technologies) was used for the transfection of siRNAs. The transfections were performed, as recommended by the manufacturers. For siRNA experiments, two consecutive transfections, 24 hours apart, were performed.

### RNA Isolation, cDNA Synthesis, and Real-Time Quantitative PCR Analysis

RNA was isolated with Trizol reagent (Life Technologies), as described by the supplier. SuperScript II Reverse Transcriptase (Life Technologies) and random hexamers (Applied Biosystems) were used for cDNA synthesis. qPCR assays using Taqman probe and primers were performed, as described previously [41].

### XBP1-splicing assay

X-box binding protein 1 (XBP1) splicing assay is based on the fact that spliced form of XBP1 mRNA lacks the recognition sequence for Pst1 enzyme, which is present in the unspliced form [42]. In brief, PCR was performed using cDNA, synthesized from HepG2 cell RNA, and a XBP1 specific primer pair: (forward: 5’-AAACAGAGTAGCAGCTCAGACTGC-3’, reverse: 5’-TCCTTCTGGGTAGACCTCTGGGAG-3’). The amplicons 448 bp and 474 bp, which indicate spliced and unspliced forms of XBP1, respectively were subsequently digested with Pst I at 37°C for 2 hours. Following digestion, unspliced XBP1 amplicon (474 bp) yielded 290 bp and 183 bp fragments. The digested products were subjected to DNA agarose gel electrophoresis. GAPDH, used as the loading control, was amplified with the following primer pair: (forward: 5’- TGGTATCGTGGAAGGACTC-3’, reverse: 5’- AGTAGAGGCAGGGATGATG-3’).

### Cell lysis and western blotting

Cells were lysed with phopho-lysis buffer [10 mM Tris-HCl (pH 7.4), 100 mM NaCl, 5 mM EDTA and 10% glycerol] supplemented with 1 mM PMSF, 0.5% Triton X-100, protease inhibitor cocktail, pepstatin A (Sigma-Aldrich), phosphatase inhibitor cocktail (Sigma), and activated sodium orthovanadate (Acros Organics). Three rounds of 5 sec. sonications at 50% amplitude with ultrasonic dismembrator 150T (Fisher Scientific) were performed to facilitate cell lysis. After removing cell debris by centrifugation (3000 x g for 5 min.), supernatants were used for western blotting.

Western blotting was performed, as described previously [41]. Primary antibodies, anti-Phospho-STAT3 (Tyr 705) (Cell signaling #9131), anti-STAT3 (#9132), anti-Phospho-JNK (#9251), anti-JNK (9252), anti-Phospho-NF-κB p65 (#3033), anti-NF-κB p65 (#4764), anti-GRP78 (Santa Cruz, # SC-166490), anti-CHOP (#SC-7351), anti-gapdh (Millipore, #MAB374), and secondary anti-mouse or anti-rabbit antibodies (Cell signaling) were obtained commercially. Immune-reactive bands were detected by the ImmunStar-AP Substrate (Bio-Rad Laboratories).

### Plasmid DNA constructs, mutagenesis and dual luciferase reporter assays

For cloning, the 0.6 kb *HAMP* promoter region (+36 to -593) was amplified by PCR using Phusion High-Fidelity DNA Polymerase (Thermo Scientific) and specific primers [(Forward primer with MluI site: 5’- GGACGCGTTGTCATTTATGGCCAAAAGTTTGCT-3’) (Reverse primer with XhoI site: 5’- GACTCGAGTGAGCTTGCTCTGGTGTCTG-3’)]. Amplified and purified PCR product was digested with the corresponding restriction enzymes, and inserted into pGL3-Basic Luciferase Vector (Promega). The ligated plasmid was electroporated into electro-competent DH5α bacterial cells with MicroPulse electroporator system (Bio-Rad). The transformed bacteria were selected on LB Agar plates containing Ampicillin. Single colonies were picked and cultured for plasmid purification with a commercial miniPrep kit (Qiagen). The insert sequences of recombinant plasmids were verified by DNA sequencing. For transfections, plasmids purified by a MaxiPrep kit (Qiagen) were used.

Mutation of the consensus STAT3 DNA-binding site on the 0.6 kb *HAMP* promoter region was performed with Quickchange II Site-Directed Mutagenesis kit (Agilent Technologies) using specific primers: (forward: 5'-CTGTCTCATTTCCAGGTGGTGGCGCCGAAAA-3' reverse: 5'-TTTTCGGCGCCACCACCTGGAAATGAGACAG-3'), as described by the manufacturer.

HepG2 cells were transfected with either empty pGL-3 Basic vector (as control) or recombinant reporter vector, as described. pRL-SV40 plasmid was co-transfected as a reference for transfection efficiency. Dual luciferase reporter assays were performed according to the manufacturer’s guidelines (Promega).

### Promoter analysis and chromatin immunoprecipitation (ChIP) assays

The presence of consensus DNA-binding sites for transcription factors, STAT3 and AP-1 within the 0.6 kb *HAMP* promoter region have previously been identified [43,44]. We have identified a putative NF-κB (subunit P65) binding site on 0.6kbp *HAMP* promoter (-583bp to -592bp from ATG) by using MatInspector [45]. ChIP assays were performed, as described previously [46]. Briefly, 50 x10^6^ HepG2 cells were fixed with 1% formaldehyde for 10 min. at room temperature. The cross-linking was stopped with 0.1375M glycine solution. Cells, washed twice with cold 1xPBS, were subsequently lysed with ChIP lysis buffer [5 mM PIPES (pH 8.0), 85 mM KCl, 0.5% Nonidet P-40 (NP-40)] supplemented with protease inhibitors. Nuclei fractions collected by centrifugation at 1000 x g for 10 minutes at 4°C, were lysed on ice for 10 minutes with a lysis buffer [50 mM Tris-Cl (pH 8.0), 10 mM EDTA, 1% SDS, protease inhibitors]. The lysates were then sonicated using ultrasonic dismembrator 150T (Fisher Scientific) with the following settings: (50% amplitude, 6 x 30 sec. cycles). 100 μg of chromatin was pre-cleared for 2 hours at 4°C with 50 μl protein A-Agarose beads (Santa Cruz) blocked with Herring Sperm DNA (Promega). 5 μl of the pre-cleared chromatin was saved as input control. The pre-cleared chromatin was then incubated overnight at 4°C with anti-STAT3 (Cell signaling, #9132), anti-NF-κB p65 (#4764), anti-c-Jun (Santa Cruz, #sc-45) antibodies or normal rabbit/mouse IgG (Santa Cruz), as a negative control. The next day, chromatin and IgG complexes were captured by incubating with 50 μl of blocked protein A-Agarose beads for 2 hours at 4°C. Immunoprecipitates were subjected to sequential washes as follows: (1x with low salt buffer [20 mM Tris/HCl (pH 8.0), 2 mM EDTA, 150 mM NaCl, 0.1% SDS and 1% Triton X-100], 1 X with high salt buffer [20 mM Tris/HCl (pH 8.0), 2 mM EDTA, 500 mM NaCl, 0.1% SDS and 1% Triton X-100] and 1 X with lithium chloride wash buffer [10 mM Tris/HCl (pH 8.0), 1 mM EDTA, 250 mM LiCl, 1% Triton X-100, 1% Igepal and 1% deoxycholic acid], and 3x with 1x TE buffer. Washed chromatin was subsequently eluted using a freshly-prepared elution buffer (100 mM NaHCO_3_ and 1% SDS), and reverse cross-linked with 200 mM NaCl at 65°C overnight. The next day, samples were treated with DNase-free RNase and proteinase K (Fisher Scientific), and purified using a commercial Qiaquick PCR purification kit (Qiagen). *HAMP* promoter was amplified by PCR using specific primers designed with Primer-BLAST (NCBI), as listed in Table 1.

**Table 1:** The sequence of primers used for ChIP assays.

Gene	Forward Primer	Reverse Primer
STAT3	GAGGGTGACACAACCCTGTT	ACCGAGTGACAGTCGCTTTT
NF-κB	TCATTTATGGCCAAAAGTTTGCT	CAAGCATCAGCGTGTGCC
c-Jun	TGAGGGTGACACAACCCTGT	CTGCTGGGTCTTGAGCTTGC

### Statistical Analysis

The significance of difference between groups was determined by Student’s t-test or one-way ANOVA with Tukey's HSD post-hoc test by using SPSS software. A value of P < 0.05 was accepted as statistically significant.

## Results

In order to study the role of ceramide in the regulation of *HAMP* expression, human hepatoma HepG2 cells were treated with synthetic cell-permeable ceramide analogs, C2 and C6 ceramide. These analogs are commonly used to study the effects of ceramide in different experimental systems [23,39,47]. We chose 30 μM and 60 μM ceramide analog concentrations because they are widely used in other studies [33,48] and our preliminary studies have also shown that cells treated with these concentrations for 8 hours exhibit over 90% cell viability. Similar ceramide concentrations have also been detected in the plasma of human NAFLD patients [27] and in lipid-infused rats [49]. We treated control cells with solvent (0.1% DMSO).

**Fig 1:**
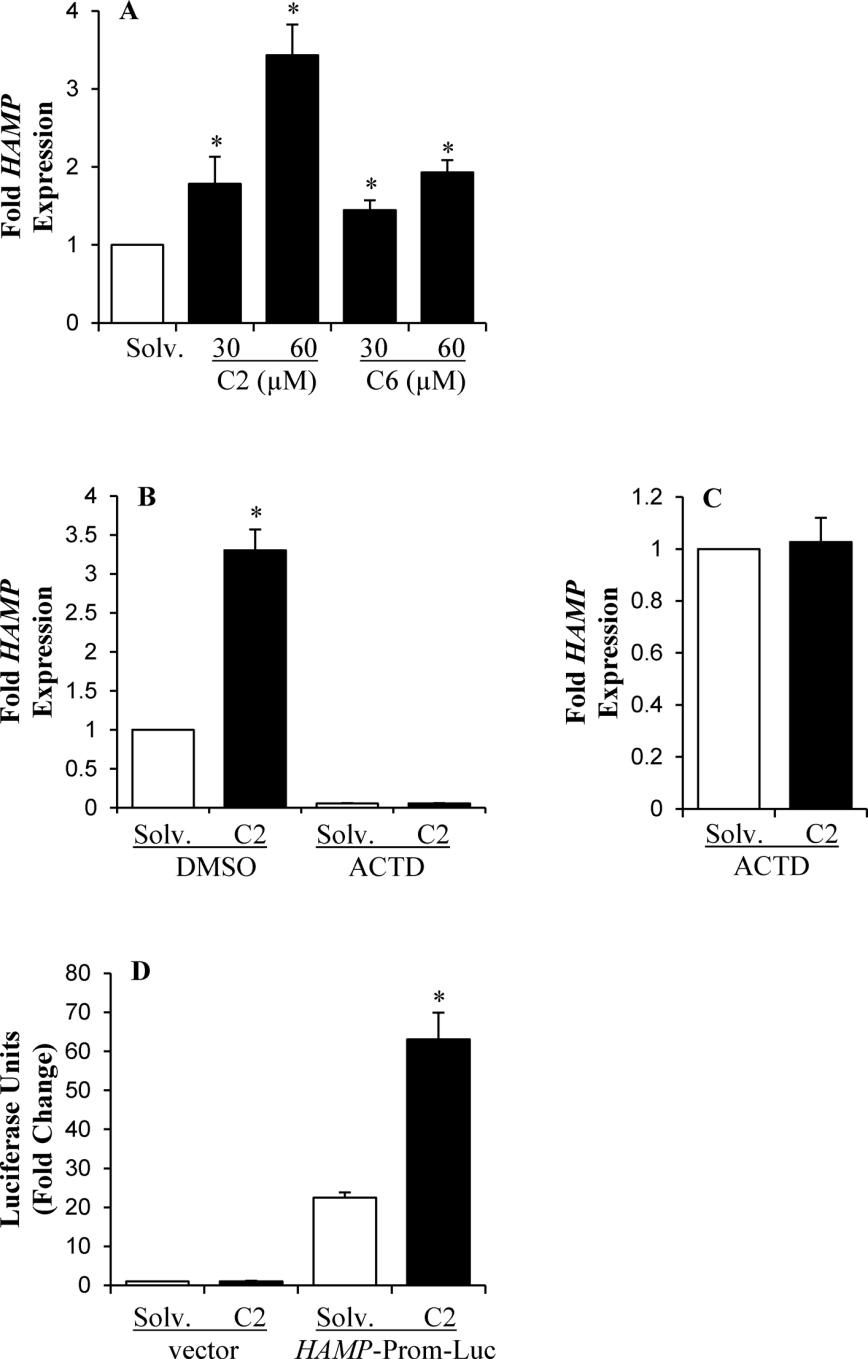
Ceramide analogs induced *HAMP* transcription. (**A**) *HAMP* mRNA expression in HepG2 cells, treated with 30 μM or 60 μM of C2 and C6 ceramide or solvent (solv.) control for 8 hours, was determined by Taqman qPCR, as described in experimental procedures. *HAMP* mRNA levels in ceramide-treated cells was expressed as fold change of that in solvent-treated cells. (**B**) HepG2 cells were treated with 60 μM C2 ceramide or solvent in the presence of either 1μg/ml actinomycin D (ACTD) or DMSO as control. *HAMP* mRNA expression, determined by qPCR, in treated cells was calculated as fold change of that in control cells, incubated with solvent and DMSO. (**C**) The effect of ACTD on *HAMP* up-regulation induced by ceramide was shown by calculating *HAMP* expression in C2 ceramide-treated cells as fold change of that in cells treated with solvent. (**D**) HepG2 cells, transfected with pGL-3 basic vector harboring 0.6kbp *HAMP* promoter (*HAMP*-Prom-Luc) or empty pGL-3 basic vector (vector), and pRL-SV40 plasmid as control for transfection efficiency, were treated with 60 μM C2 ceramide or solvent for 8 hours. Dual luciferase report assays were performed, as described in the experimental procedures. *HAMP* promoter activity, expressed in relative luciferase units, was calculated as fold change of that in cells transfected with the empty vector and treated with solvent. Asterisks indicate statistical significance (P<0.05).

The effect of ceramide analogs on hepcidin expression was determined by measuring the level of *HAMP* mRNA by qPCR, as described in the experimental procedures. HepG2 cells treated with 30 μM or 60 μM of C2 ceramide displayed an 1.78 ± 0.35 and 3.43 ± 0.39 -fold increase in *HAMP* expression, respectively, compared to control cells treated with solvent (**Fig 1A**). Both C2 and C6 ceramide analogs significantly elevated *HAMP* mRNA expression (**Fig 1A**). Since, C2 exerted a more potent effect than C6 under similar experimental conditions, we employed C2 ceramide analog for further experiments.

Studies to this date have shown that *HAMP* expression is regulated via transcription [44,50–52]. To determine whether ceramide also regulated *HAMP* at the transcriptional level, we employed the transcription inhibitor, actinomycin D. For these experiments, HepG2 cells were treated with ceramide or solvent in the presence of either actinomycin D or DMSO, as control. The addition of DMSO did not alter C2 ceramide-mediated induction of *HAMP* mRNA expression (**Fig 1B**). Actinomycin D by itself significantly decreased the level of *HAMP* mRNA in HepG2 cells (**Fig 1B**), Simultaneous treatment of HepG2 cells with actinomycin D and ceramide completely blocked ceramide-induced *HAMP* mRNA up-regulation (**Fig 1B and 1C**). To further confirm the involvement of transcriptional mechanisms in *HAMP* regulation by ceramide, reporter assays were performed using a 0.6 kb region of *HAMP* promoter, which harbors binding sites for various transcription factors [43,52] (**Fig 1D**). HepG2 cells, transfected with either empty pGL-3 basic vector or vector containing 0.6kb *HAMP* promoter (*HAMP*-Prom-Luc), were co-transfected with pRL-SV40 plasmid encoding renila luciferase as a reference for transfection efficiency, as described in experimental procedures. A significantly higher level of luciferase activity was detected in cells transfected with the vector harboring *HAMP* promoter compared to cells transfected with the empty pGL-3 basic vector (**Fig 1D**). Ceramide treatment further increased reporter activity in HepG2 cells transfected with *HAMP*-Prom-Luc, but not with empty vector (**Fig 1D**). Both actinomycin D and reporter assay studies have shown that ceramide activates *HAMP* transcription in HepG2 cells, and that this activation is conveyed through cis-elements contained within the 0.6 kb *HAMP* promoter region.

**Fig 2:**
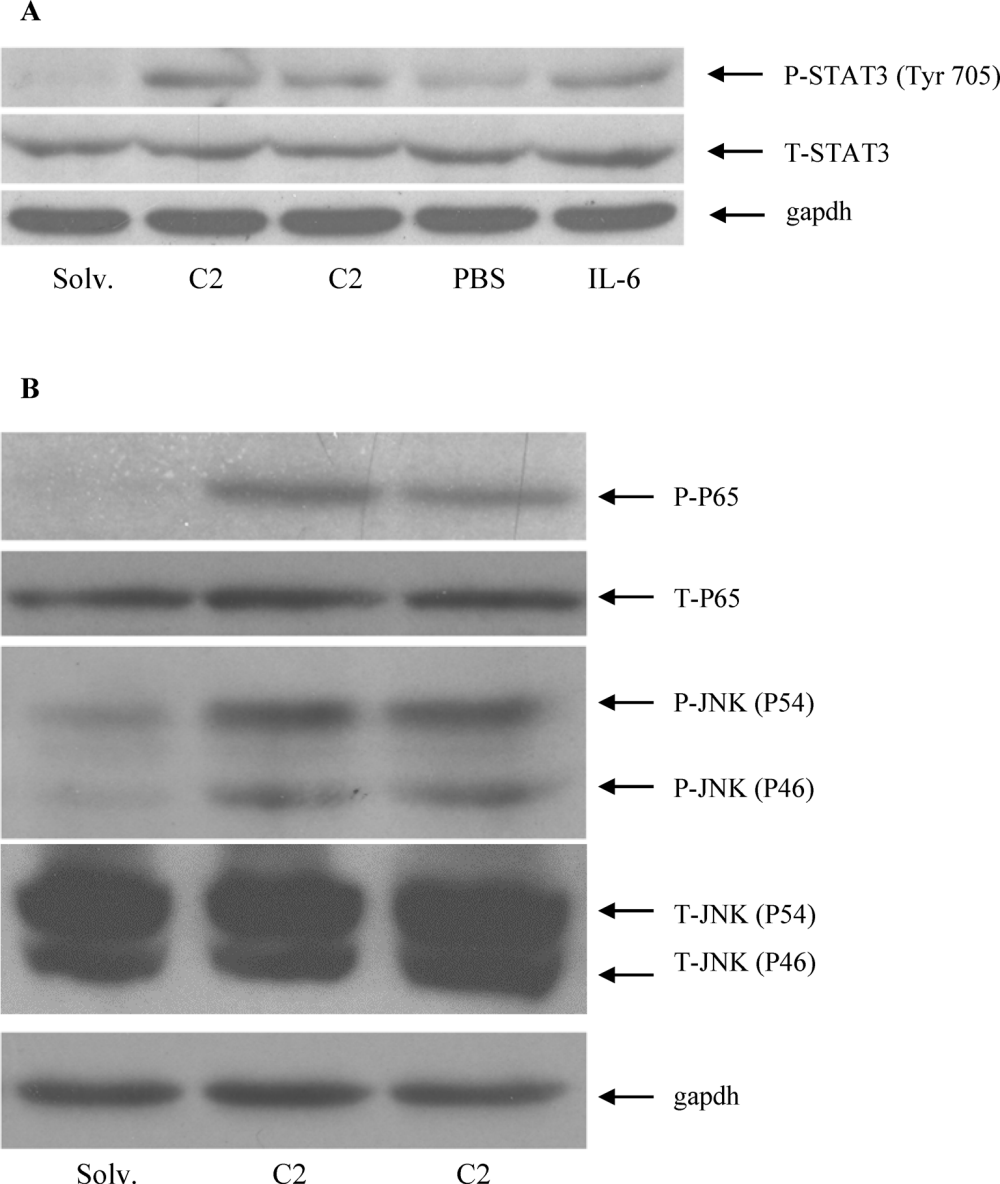
The effect of ceramide on the phosphorylation of STAT3, JNK and NF-κB in HepG2 cells. Protein lysates isolated from HepG2 cells treated with 60 μM C2 ceramide or solvent control, and 40 ng/ ml recombinant IL-6 or PBS control for 8 hours, were employed for western blotting to determine the level of total or phosphorylated proteins. Total STAT3 (T-STAT3) or STAT3 phosphorylated on tyrosine 705 (P-STAT3) (**A**), total (T-P65) or phosphorylated (P-P65) NF-κB subunit P65, and total (T-JNK) or phosphorylated-JNK (P-JNK) (**B**) protein levels were determined by using specific antibodies. An anti-gapdh antibody was employed as a protein loading control.

Ceramide treatment has been reported to induce the activation of various inflammatory signaling pathways including JAK/STAT3, NF-κB and JNK/c-Jun *in vitro* [31–34,38–40]. However, its signaling effect in hepatoma cells is largely unknown. Therefore, western blots were performed using specific anti-phospho antibodies to examine the activating status of these signaling molecules (**Fig 2A and 2B**). STAT3 is phosphorylated on tyrosine (Tyr 705) residue by JAK kinases for activation [53]. Ceramide significantly stimulated the tyrosine phosphorylation of STAT3 in HepG2 cells (**Fig 2A**). HepG2 cells treated with recombinant IL-6, as a positive control, also displayed STAT3 phosphorylation at tyrosine 705, confirming the specificity and sensitivity of our antibody and western blotting assays (**Fig 2A**). The expression level of total STAT3 protein in HepG2 cells was not altered by ceramide or IL-6 (**Fig 2A**). Similar to STAT3, ceramide also stimulated the phosphorylation of NF-κB subunit P65 and JNK (**Fig 2B**) in HepG2 cells.

**Fig 3:**
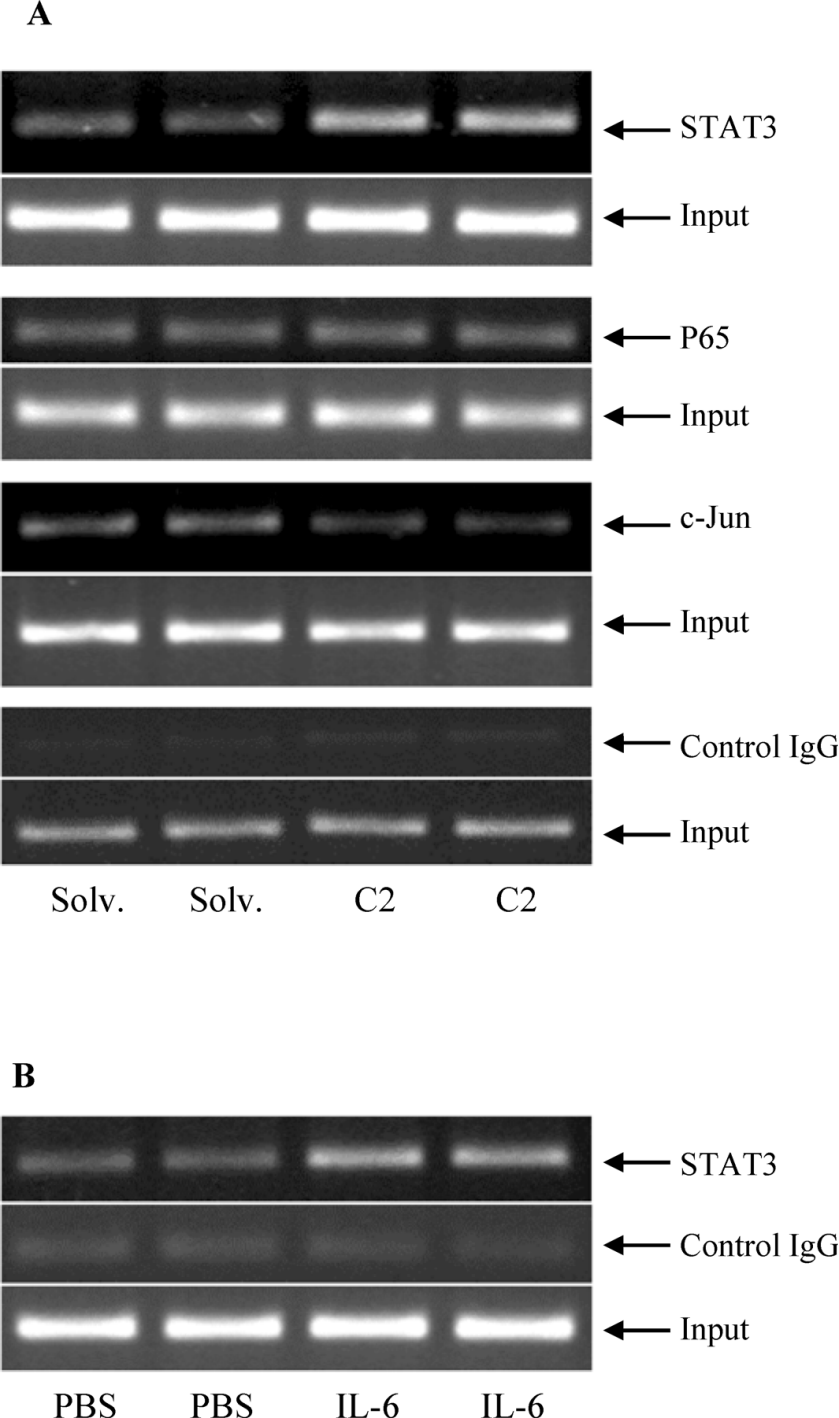
Ceramide stimulated the binding of STAT3, but not NF-κB subunit P65 or c-Jun, to *HAMP* promoter. (**A**) Chromatin, isolated from HepG2 cells treated with 60 μM C2 ceramide or solvent (solv.) for 8 hours and fixed with 1% formaldehyde, was incubated with antibodies specific for STAT3, NF-κB subunit P65 or c-Jun for chromatin immunoprecipitation (ChIP) assays, as described in the experimental procedures. Normal rabbit or mouse IgG were employed as negative controls. Immunoprecipitated and purified chromatin and total input chromatin (loading control) were used for PCR to amplify a *HAMP* promoter region harboring corresponding consensus sequences by using specific primers. Representative images of amplicons, analyzed by DNA agarose gel electrophoresis, visualized by ethidium bromide staining, and captured using Gel Doc XR+ system (Bio-Rad), are shown. (**B**) ChIP assays were performed with HepG2 cells treated with recombinant IL-6 or PBS (control) by using anti-STAT3 antibodies, as described above.

In order to determine the role of ceramide-induced signaling pathways in *HAMP* transcriptional activation, various experiments were performed. Chromatin immunoprecipitation (ChIP) assays were utilized to identify the transcription factors physically interacting with *HAMP* promoter in ceramide-treated HepG2 cells (**Fig 3A and 3B**). Specific commercial antibodies validated for ChIP assay were employed to detect the binding of transcription factors, STAT3, NF-κB and c-Jun to *HAMP* promoter. Same concentrations of normal rabbit or mouse IgG were used as negative controls. The primers used for ChIP analysis (**Table 1**) amplified *HAMP* promoter regions, which harbored consensus sites for respective transcription factors (STAT3: -135bp to -143bp; AP-1: -126bp to -134bp; NF-κB P65: -583bp to -592bp proximal to the start codon, ATG). ChIP assay results indicated that ceramide significantly increased the binding of STAT3 to *HAMP* promoter, compared to solvent treatment in HepG2 cells (**Fig 3A**). As a positive control, HepG2 cells were treated with the cytokine, IL-6, a well-characterized inducer of *HAMP* transcription through STAT3 activation [15]. IL-6 stimulated the binding of STAT3 to *HAMP* promoter, which validated our ChIP assay system (**Fig 3B**). Unlike STAT3, ceramide did not activate the binding of NF-κB p65 subunit to *HAMP* promoter (**Fig 3A**). In contrast, the binding of c-Jun, a classical transcription factor activated by JNK, to *HAMP* promoter was weaker in ceramide-treated HepG2 cells compared to that in solvent-treated cells (**Fig 3A**). Our ChIP results were specific because no significant immune precipitation was observed with control IgG samples in any of our ChIP assays (representative images are shown in **Fig 3A and 3B**). Furthermore, equal amount of chromatin was used across the samples in our ChIP assays, as shown by the total amount of chromatin input (**Fig 3A and 3B**).

**Fig 4:**
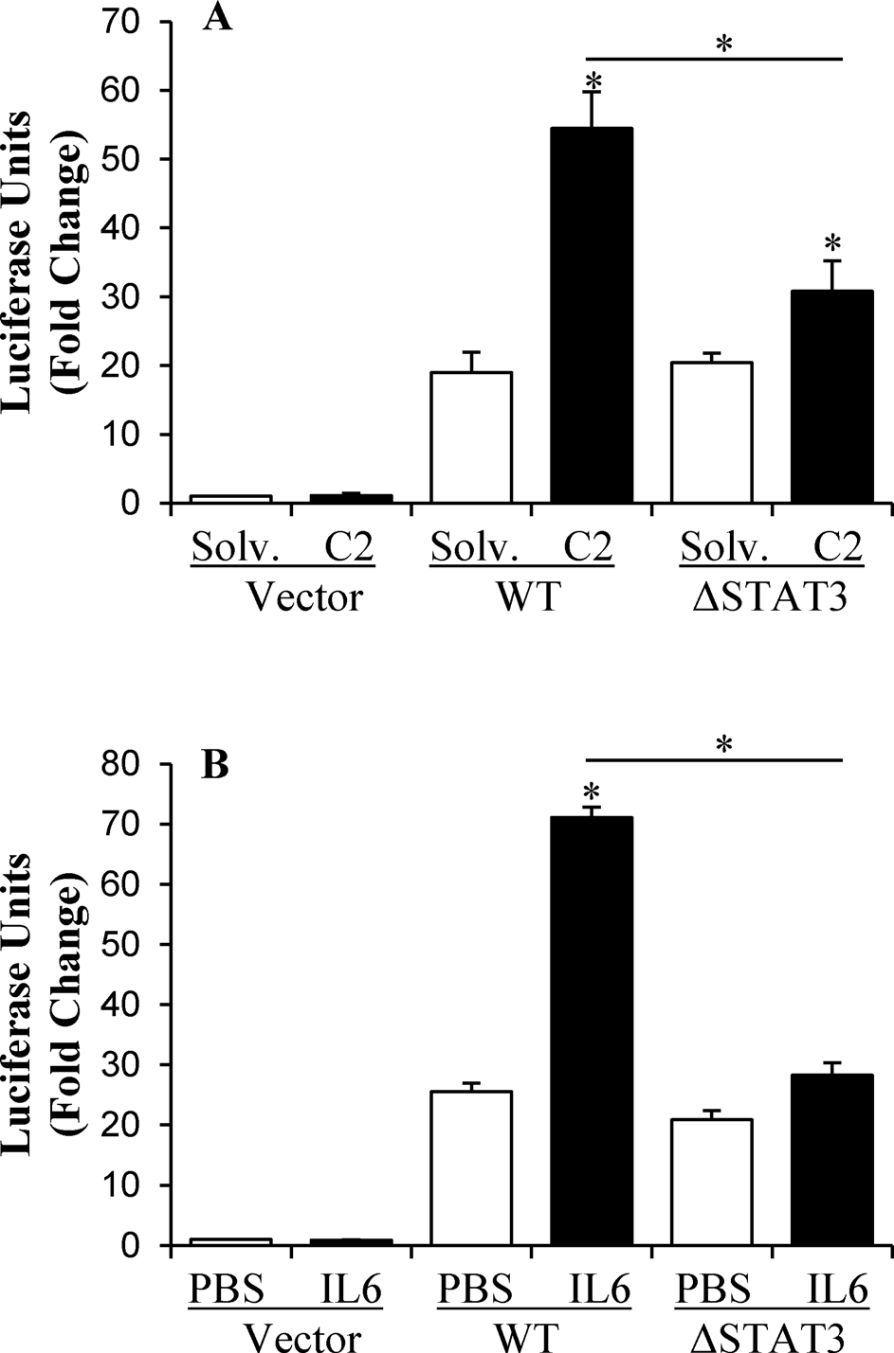
The effect of STAT3 response element mutation on *HAMP* promoter activation by ceramide. The single STAT3 response element within 0.6kbp *HAMP* promoter region cloned in pGL3 basic vector was mutated, as described in the experimental procedures. HepG2 cells, transfected with pGL3-basic vector harboring wild-type (WT) or mutated (ΔSTAT3) 0.6 kb *HAMP* promoter, or empty vector (vector) as negative control, and pRL-SV40 plasmid, as control for transfection efficiency, were treated with (**A**) 60 μM C2 ceramide (C2) and solvent control (solv.) or (**B**) 40 ng/ ml IL-6 and PBS control for 8 hours. Dual luciferase reporter assays were performed, as described in the experimental procedures. *HAMP* promoter activity, expressed in relative luciferase units, in treated cells was calculated as fold change of that in control cells transfected with empty vector-and treated with solvent. Asterisks indicate statistical significance (P<0.05).

To validate the direct involvement of STAT3 in ceramide-mediated transcriptional regulation of *HAMP*, the STAT3 response element in the 0.6kbp *HAMP* promoter reporter construct was mutated, as described in the experimental procedures. Luciferase reporter assays were then performed with pGL-3 basic vectors harboring the wild-type or mutated *HAMP* promoter (**Fig 4A**). Mutation of the STAT3 response element (ΔSTAT3) significantly diminished the stimulatory effect of ceramide on *HAMP* promoter, confirming the direct involvement of STAT3 (**Fig 4A**). We validated the mutated promoter construct with IL-6 treatment because this STAT3 response element is required for IL-6-responsiveness of *HAMP* promoter [15,44]. Accordingly, the mutation of STAT3 response element (ΔSTAT3) abolished IL-6-induced *HAMP* promoter activation (**Fig 4B**).

In order to study the involvement of JAK/STAT3 signaling in the transcriptional regulation of *HAMP* expression induced by ceramide, STAT3 siRNA and a chemical pan-JAK kinase inhibitor, JAK inhibitor I were employed (**S1A and S1B Fig**). The specificity of STAT3 siRNA was confirmed by real-time PCR and western blotting. The levels of STAT3 mRNA (**S1A Fig**) and protein (**S1A Fig inset**) expression were significantly inhibited by STAT3 siRNA as compared to control siRNA-transfected HepG2 cells. JAK inhibitor I treatment significantly inhibited ceramide-induced phosphorylation of STAT3 at residue Tyr 705 under our experimental conditions, as confirmed by western blotting (**S1B Fig**).

**Fig 5:**
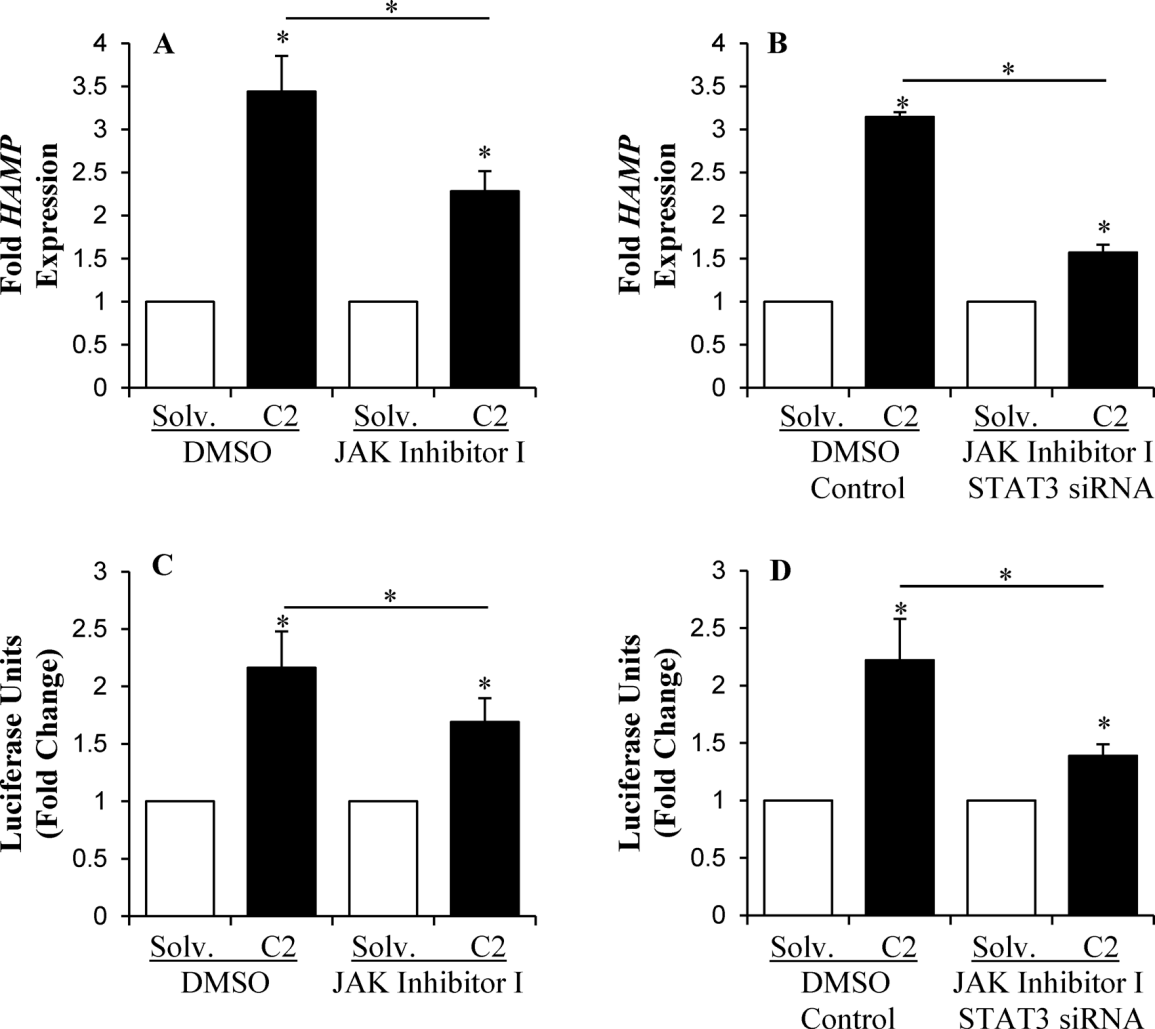
Activation of STAT3 signaling was required for the induction of *HAMP* transcription by ceramide (A) HAMP mRNA expression in HepG2 cells, treated with solvent (solv.) or C2 ceramide for 8 hours in the presence of either 5 μM JAK inhibitor I or DMSO (control), was determined by Taqman qPCR. HAMP expression in treated cells was calculated as fold change of that in respective control cells treated with solvent. **(B)** HepG2 cells, pre-transfected with STAT3 or control siRNA, were treated with C2 ceramide or solvent (solv.) in the presence of 5 μM JAK inhibitor I or DMSO. HAMP mRNA levels, determined by qPCR, were expressed as fold expression of that in respective controls treated with solvent. **(C)** HepG2 cells, transfected with pGL3-basic vector harboring 0.6 kb HAMP promoter, were treated with C2 ceramide or solvent (solv.) for 8 hours in the presence or absence of 5 μM JAK inhibitor I. Dual luciferase assays were performed and HAMP promoter activity, calculated as fold change of that in corresponding control cells treated with solvent, was expressed in relative luciferase units. **(D)** HepG2 cells, pre-transfected with STAT3 or control siRNA, were subsequently transfected with pGL3-basic vector harboring 0.6 kb wild-type HAMP promoter. After treatment with solvent (solv.) and C2 ceramide in the presence of either JAK inhibitor I or DMSO, dual luciferase reporter assays were performed. *HAMP* promoter activity, expressed as relative luciferase units, was expressed as fold change of that in respective control cells treated with solvent. Asterisks indicate statistical significance (P<0.05).

The effect of JAK inhibitor I on the regulation of *HAMP* mRNA expression by ceramide was determined by qPCR (**Fig 5A**). The addition of DMSO, which is the vehicle for JAK inhibitor I did not affect ceramide-induced *HAMP* up-regulation (3.44 ± 0.41 fold). In comparison, ceramide-mediated induction of *HAMP* expression was significantly attenuated by JAK inhibitor I (2.28 ± 0.23 fold) (**Fig 5A**). Since the inhibition was incomplete, HepG2 cells were further pre-treated with both STAT3 siRNA and JAK inhibitor I. Control cells were administered with control siRNA and DMSO in parallel. Both groups of pre-treated cells were then subjected to either ceramide or DMSO (control) treatment and *HAMP* mRNA expression was determined **(Fig 5B)**. Control siRNA and DMSO pre-treatment did not alter the effect of ceramide on *HAMP* mRNA expression levels (3.15 ± 0.05 fold increase **Fig 5B**). By contrast, the combined pre-treatment with STAT3 siRNA and JAK inhibitor I decreased the induction of *HAMP* mRNA expression by ceramide (1.57 ± 0.09 fold increase **Fig 5B**). The effect of JAK inhibitor I and STAT3 siRNA on ceramide-induced *HAMP* promoter activation was further examined with luciferase reporter assays (**Fig 5C and 5D**). HepG2 cells, transfected with pGL-3 basic vector harboring 0.6 kbp *HAMP* promoter, were treated with ceramide or solvent in the presence of either JAK inhibitor I or DMSO as control. Ceramide treatment induced a 2.16 ± 0.32 fold increase in *HAMP* promoter activity, as compared to control cells treated with solvent in the presence of DMSO. In contrast to DMSO, the addition of JAK inhibitor I significantly diminished ceramide-induced *HAMP* promoter activation (1.69 ± 0.21 fold) (**Fig 5C**). The level of inhibition was stronger with the combined pre-treatment of STAT3 siRNA and JAK inhibitor I (1.39 ± 0.10 fold) (**Fig 5D**). HepG2 cells pre-treated with control siRNA and DMSO displayed 2.22 ± 0.36 fold increase in *HAMP* promoter activity following ceramide treatment (**Fig 5D**).

**Fig 6:**
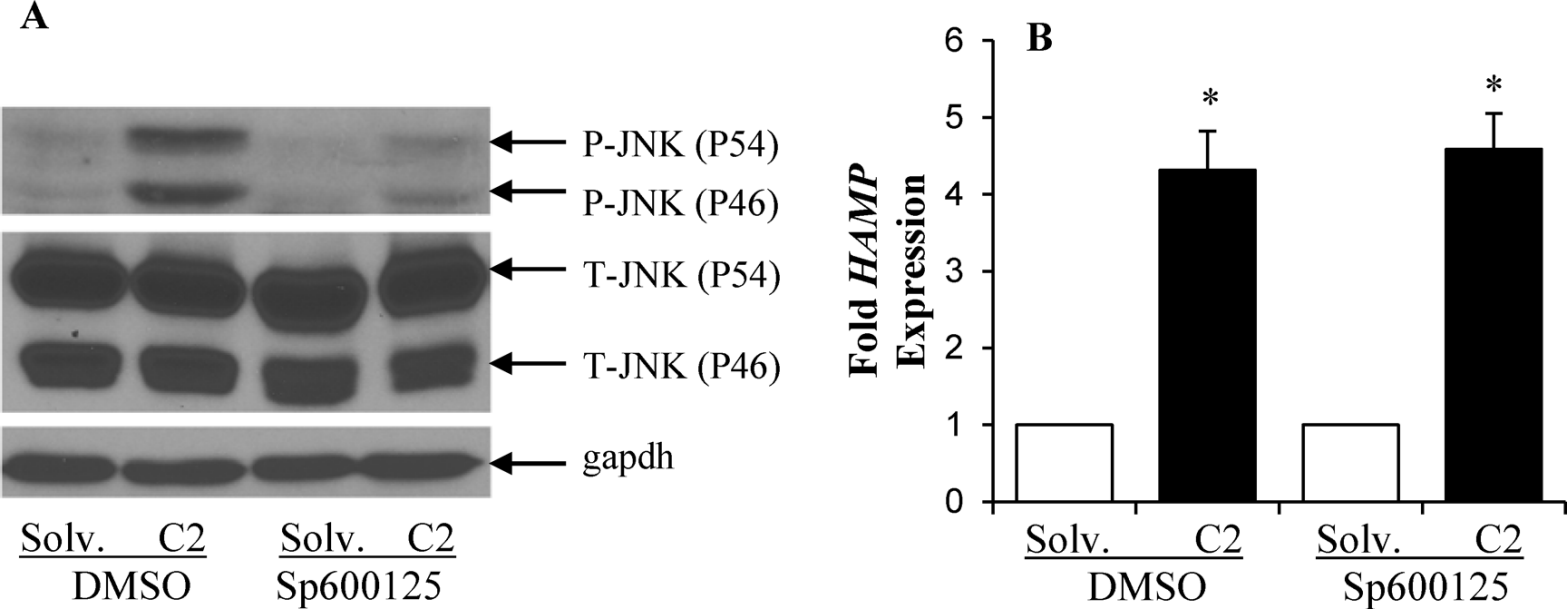
Ceramide-induced *HAMP* up-regulation was not dependent on c-Jun N-terminal kinase (JNK) phosphorylation. HepG2 cells treated with ceramide or solvent in the presence of 50 μM chemical JNK inhibitor, Sp600125 or DMSO (control) were used to isolate protein (**A**) for western blotting to determine phosphorylated (P-JNK) and total JNK protein (T-JNK) levels using specific antibodies or to isolate RNA (**B**) to determine *HAMP* mRNA expression by Taqman qPCR. *HAMP* expression in ceramide-treated cells was calculated as fold expression of that in respective control cells treated with solvent. Asterisks indicate statistical significance (P<0.05).

The mutation of STAT3 response element or the inhibition of STAT3 did not completely eliminate the stimulatory effect of ceramide on *HAMP* promoter activity and mRNA expression. The residual activation may be facilitated by other signaling pathways stimulated by ceramide. We therefore examined the involvement of JNK and ER stress, which are activated by ceramide. To study the effect of ceramide-induced JNK activation on *HAMP* expression, we employed SP600125 a specific chemical inhibitor of JNK activation [54]. HepG2 cells were treated with ceramide in the presence of SP600125 or DMSO, as control. The inhibition of ceramide-induced JNK phosphorylation by SP600125 was confirmed by western blotting using anti-phospho-JNK antibodies (**Fig 6A**). DMSO did not exert any specific effect on JNK phosphorylation (**Fig 6A**). Ceramide-induced phosphorylation of JNK was significantly blocked by SP600125 treatment under our experimental conditions (**Fig 6A**). The effect of SP600125 on ceramide-mediated increase in *HAMP* mRNA expression levels was examined by qPCR. Despite inhibiting JNK phosphorylation, SP600125 did not interfere with the induction of *HAMP* mRNA by ceramide treatment in HepG2 cells (**Fig 6B**). These results exclude a role of JNK in the up-regulation of *HAMP* expression by ceramide.

**Fig 7:**
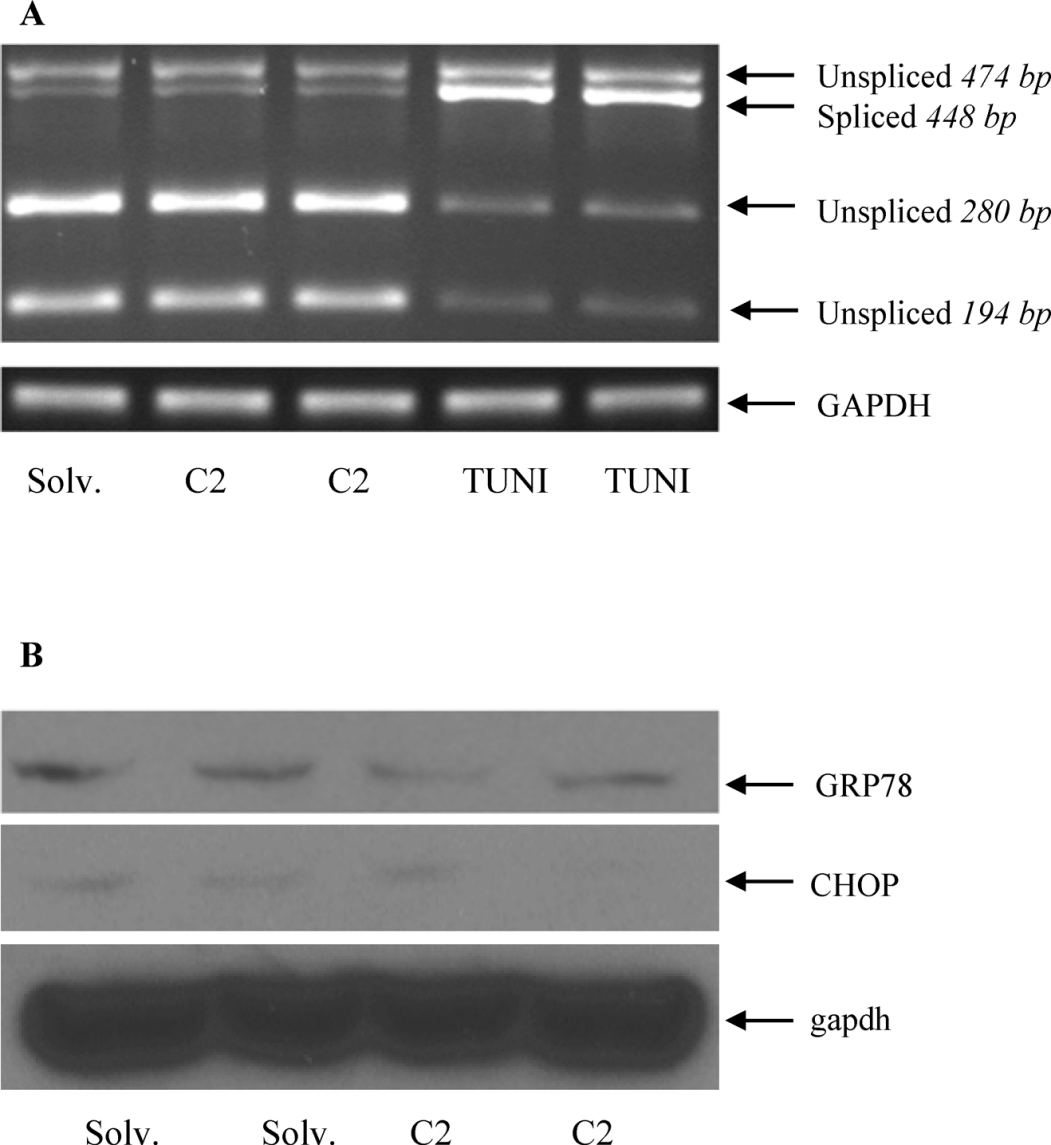
Ceramide did not induce ER stress in HepG2 cells (A) HepG2 cells, treated with 60 μM C2 ceramide and solvent (solv.) control or 10 μg/ml tunicamycin (TUNI), as positive control, for 8 hours were used for XBP1 splicing assay, as described in the experimental procedures. **(B)** Whole cell lysates from solvent (solv.) or ceramide-treated HepG2 cells were employed for western blotting to determine GRP78 and CHOP protein expression using specific antibodies. An anti-gapdh antibody was employed as a protein loading control.

Ceramide has been shown to trigger ER stress in neuronal cells and various cancer cell lines [55–57] but not in HepG2 cells. Independent studies have also reported activation of hepatic *HAMP* transcription by ER stress [58]. We determined the effect of ceramide on ER stress in HepG2 cells by XBP1-splicing assay, as described in experimental procedures. Spliced form of XBP1 was employed as an ER stress marker because ER stress-activated inositol-requiring enzyme 1 (IRE1) is known to splice X-box binding protein 1 (XBP1) mRNA [59]. Tunicamycin, a well-known chemical ER stress inducer [60], was also used as a positive control. Tunicamycin significantly increased the spliced and decreased the unspliced forms of XBP1, confirming the validity of our assay (**Fig 7A**). In contrast, ceramide treatment of HepG2 cells did not induce any significant changes in XBP1 splicing compared to solvent-treated control cells (**Fig 7A**). Furthermore, ceramide did not alter the protein expression of GRP78 and CHOP, known markers of ER stress activation, supporting the conclusion that ER stress is not activated by ceramide in HepG2 cells (**Fig 7B**).

## Discussion

Both inflammation and dysregulation of iron homeostasis correlate with disease severity in NAFLD patients [5–7,61]. Since hepcidin is both an acute phase and iron-regulatory protein, and its expression is modulated in NAFLD, it is important to understand *HAMP* regulation by different lipids. Here, we have shown a specific role for the lipid species, ceramide in the regulation of *HAMP* transcription via the activation of JAK/STAT3 signaling in human hepatoma cells.

Besides actinomycin D, our promoter assays also indicated the effect of ceramide on *HAMP* transcription, which was confined to a 0.6kbp promoter region proximal to the translation initiation site. The STAT3 response element, harbored within this region of *HAMP* promoter, has previously been reported to mediate the transcriptional regulation of *HAMP* by IL-6 [15,44]. It is however unknown whether the activation of JAK/STAT3 signaling by other stimuli such as ceramide also exerts similar effects on *HAMP* transcription. Our findings in this study confirmed the presence of such a mechanism in human hepatoma cells. Firstly, ceramide induced the phosphorylation of STAT3 on tyrosine 705, and its interaction with *HAMP* promoter in HepG2 cells. Secondly, the mutation of this particular STAT3 response element significantly inhibited the stimulatory effect of ceramide on *HAMP* promoter activity, indicating the requirement for STAT3 binding to *HAMP* promoter for the activation of *HAMP* transcription by ceramide. Furthermore, inhibition of STAT3 with a chemical inhibitor and siRNA significantly decreased the stimulatory effect of ceramide on *HAMP* promoter activity and mRNA expression.

Although we have established a novel role for ceramide in hepcidin regulation, it is also important to note that this may not be entirely mediated by JAK/STAT3 signaling. Namely, the positive effect of ceramide on hepcidin mRNA expression and promoter activity was not completely blocked by the inhibition of JAK/STAT3 signaling. Moreover, despite the mutation of STAT3 response element within 0.6 kb *HAMP* promoter, a residual activation of *HAMP* promoter was still observed in ceramide-treated HepG2 cells. On the other hand, this mutation completely reversed the effect of IL-6. These results indicate that unlike IL-6, ceramide may not be completely dependent on STAT3 for *HAMP* promoter activation, and utilizes additional signaling pathways. Accordingly, we have shown the stimulation of NF-κB and JNK phosphorylation by ceramide in HepG2 cells. Similar effects were also reported in other cells [32,33,38–40]. Despite elevated phosphorylation, our ChIP assays did not detect changes in NF-κB binding to *HAMP* promoter. Interestingly, ceramide weakened the basal level of c-Jun binding to *HAMP* promoter. This negative effect does not involve JNK because ceramide stimulated JNK phosphorylation in HepG2 cells. It may be due to a potential competition with STAT3 because their response elements are in close proximity within 0.6kb *HAMP* promoter region (i.e. AP-1: -125 to -134; STAT3: -135 to -143 proximal to ATG). Additionally, our results with SP600125, which inhibited JNK phosphorylation but did not alter ceramide-induced *HAMP* expression, also exclude a role for JNK/c-Jun signaling in the regulation of *HAMP* transcription by ceramide.

Aberrant lipid metabolism in animal models of obesity [62] and treatment of human cancer cells with ceramide [55,57] have been shown to trigger ER stress. Experimental ER stress inducers are known to activate *HAMP* transcription [58]. However, ceramide failed to induce ER stress in HepG2 cells under our experimental conditions. Similarly, a role for ceramide was excluded in ER stress induced by saturated fatty acids in liver cells [63]. Although NF-κB, JNK/c-Jun or ER stress are not involved, this does not exclude a role for other pathways, which may potentially act in synergy with JAK/STAT3 signaling to mediate the activation of *HAMP* transcription by ceramide in liver cells.

In summary, we have demonstrated a novel role for the biologically active lipid intermediate, ceramide in the transcriptional regulation of *HAMP* in human liver cells. Our findings have also indicated that ceramide can directly activate inflammatory signaling in hepatocytes *in vitro* in the absence of macrophages or cytokines. This study establishes an important link between lipid-mediated inflammation and iron metabolism, which may have implications for fatty liver disease pathology and obesity. The involvement of ceramide and hepcidin in NAFLD disease progression warrants further research.

## Supporting Information

S1 Fig STAT3 protein and mRNA expression, and ceramide-induced activation of STAT3 were inhibited by STAT3 siRNA and JAK inhibitor I, respectively. (DOCX)
