# Supplementary material for: Ceramide Induces Human Hepcidin Gene Transcription through JAK/STAT3 Pathway
Source: PLoS One. 2016 Jan 25;11(1):e0147474. doi: 10.1371/journal.pone.0147474 (PMC4726556; doi:10.1371/journal.pone.0147474)
Supplement: S1 Fig — (DOCX) [file pone.0147474.s001.docx]

**Supporting Information**

**Legends to Supplementary Figures**

**S1 Fig.** **STAT3 protein and mRNA expression, and ceramide-induced activation of STAT3 were inhibited by STAT3 siRNA and JAK inhibitor I, respectively.** (**A**) STAT3 mRNA and protein (inset) levels in HepG2 cells transfected with STAT3 siRNA or control siRNA were determined by qPCR or western blotting, respectively. Asterisks indicate statistical significance (P<0.05). (**B**) Cell lysates prepared from HepG2 cells, treated with 60 μM C2 ceramide or solvent (solv.) in the presence of 5 μM JAK inhibitor I or DMSO as control, were used for western blotting to detect total (T-STAT3) or tyrosine-phosphorylated (P-STAT3) STAT3 protein levels using specific antibodies. An antibody for gapdh was used as control for protein loading.


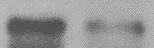


STAT3

**A**


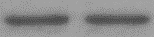


Control STAT3

siRNA

gapdh

*

Control STAT3

**B**

P-STAT3 (Tyr 705)

T-STAT3

gapdh

Solv. C2 Solv. C2

DMSO JAK Inhibitor I

**S1 Fig.**
